# Variation in Body Shape across Species and Populations in a Radiation of Diaptomid Copepods

**DOI:** 10.1371/journal.pone.0068272

**Published:** 2013-06-27

**Authors:** Stephen Hausch, Jonathan B. Shurin, Blake Matthews

**Affiliations:** 1 Department of Zoology, University of British Columbia, Vancouver, British Columbia, Canada; Consiglio Nazionale delle Ricerche (CNR), Italy

## Abstract

Inter and intra-population variation in morphological traits, such as body size and shape, provides important insights into the ecological importance of individual natural populations. The radiation of Diaptomid species (~400 species) has apparently produced little morphological differentiation other than those in secondary sexual characteristics, suggesting sexual, rather than ecological, selection has driven speciation. This evolutionary history suggests that species, and conspecific populations, would be ecologically redundant but recent work found contrasting ecosystem effects among both species and populations. This study provides the first quantification of shape variation among species, populations, and/or sexes (beyond taxonomic illustrations and body size measurements) to gain insight into the ecological differentiation of Diaptomids. Here we quantify the shape of five Diaptomid species (family Diaptomidae) from four populations each, using morphometric landmarks on the prosome, urosome, and antennae. We partition morphological variation among species, populations, and sexes, and test for phenotype-by-environment correlations to reveal possible functional consequences of shape variation. We found that intraspecific variation was 18-35% as large as interspecific variation across all measured traits. Interspecific variation in body size and relative antennae length, the two traits showing significant sexual dimorphism, were correlated with lake size and geographic location suggesting some niche differentiation between species. Observed relationships between intraspecific morphological variation and the environment suggest that divergent selection in contrasting lakes might contribute to shape differences among local populations, but confirming this requires further analyses. Our results show that although Diaptomid species differ in their reproductive traits, they also differ in other morphological traits that might indicate ecological differences among species and populations.

## Introduction

There is growing recognition that both interspecific and intraspecific variation can have significant effects on population, community, and ecosystem dynamics [1–3]. For example, Crutsinger et al. [4] found that increasing the genetic diversity of a dominant perennial plant increased both the above-ground net primary productivity and arthropod diversity. Along a similar vein, freshwater fish with contrasting foraging phenotypes within [5] and among [6] populations can drive changes in community composition of prey and ecosystem processes [7,8]. These studies illustrate that intra-specific variation and local adaptation can influence ecosystem dynamics, but also reveal that more work is needed to identify the specific phenotypes that underlie these population, community, and ecosystem effects [9].

Morphological differences are likely a key component of this ecologically important variation. An organism’s morphology is strongly associated with its growth, survival, and reproductive success in different environments [10–12] and often underlies its functional role in ecosystems [13,14]. Trade-offs among multiple traits underlying fitness can constrain an organism’s niche breadth and limit the population’s response to selective forces [15]. The differentiation of functional traits among species and populations is well studied in several classical examples of adaptive radiations, and provides insights into the nature of selection and local adaptation in natural populations. For example, the shape of finch beaks on the Galapagos varies with resource use [16], the color and life-history of Trinidadian guppies varies with predation regimes [1], and the coat color of mouse populations varies with substrate type [17].

Alongside such broad phenotype-by-environment correlations, more subtle aspects of morphological variation can also reveal the multidimensionality of niches in apparently similar habitat types [18]. Though differentiation along a single niche axis is more detectable, groups are expected to be adapted to complex combinations of resources and threats. For example, the body shape of *Anolis* lizards is roughly correlated with different microhabitats [19,20], but the specific pattern of morphological differentiation among lizard species, measured in terms of body size, body shape, head shape, lamella number, and sexual size dimorphism of lizards, reflect variation in multiple niche dimensions within each habitat [18]. The lesson from such studies is that detailed morphological analysis, even among seemingly morphologically similar species, might reveal cryptic differences in selection pressures in natural environments.

Understanding the evolutionary origins of morphological diversity is a good starting point for predicting the ecosystem-effects of organisms in nature. The importance of natural selection relative to neutral processes in governing local adaptation and differentiation among species and populations remains poorly understood [21]. Adaptive radiations often produce species that differ in traits specifically associated with acquiring resources from the environment. As such, communities that are structured by adaptive radiation have great potential for affecting ecosystem properties. By comparison, if morphological variation within and among species is not shaped by natural selection, but rather by sexual selection or neutral genetic drift, then species will likely be ecologically and functionally equivalent.

The radiation of Diaptomid copepods has been characterized by morphological stasis: there is little morphological divergence between genetically divergent species estimated to be 10-20 million years old [22]. Morphological differentiation in this radiation occurs mostly in mating traits (e.g. male reproductive structures) [23], suggesting that sexual selection may have led to reproductive isolation without any corresponding ecological differentiation [22,24]. If copepod diversification has largely been driven by divergent sexual selection with little accompanying differentiation in ecologically relevant traits, then we would expect to see few differences among species or populations in terms of their resource use, or associations between their phenotypes and environmental conditions. In a previous mesocosm experiment, we observed that different species and populations of Diaptomid copepods had contrasting effects on aquatic ecosystems, particularly in terms of their effects on primary productivity, nutrient levels, and bacterial abundance [25]. This surprising result contrasts with the apparent morphological similarity of Diaptomid species and the associated predictions of their ecological neutrality. Here we explore the possibility that subtle and previously un-quantified morphological divergence separates species and populations of Diaptomid copepods.

In this study, we first quantify shape variation among five species and four populations per a species (20 populations total) of Diaptomid copepods. We partition the morphological variation observed into components explained by species, populations, and sexes. We then test for associations between copepod morphology and lake morphometry and geographical position. Based on qualitative descriptions in taxonomic studies [22,23,26], we expected to find morphological conservatism with little variation among species or populations. To our knowledge, this is the first quantification of body shape variation among Diaptomid copepods, and so we had no a priori prediction about the level of intraspecific variation among species. However, in light of the divergent ecosystem effects of different populations of the same species [25], we expected to see some differentiation among populations in traits that might have a functional basis. Consistent with this, we were able to identify morphological traits which were associated with environmental and sex differences and varied across both species and populations.

## Methods

### Specimen Collection

**Figure 1:**
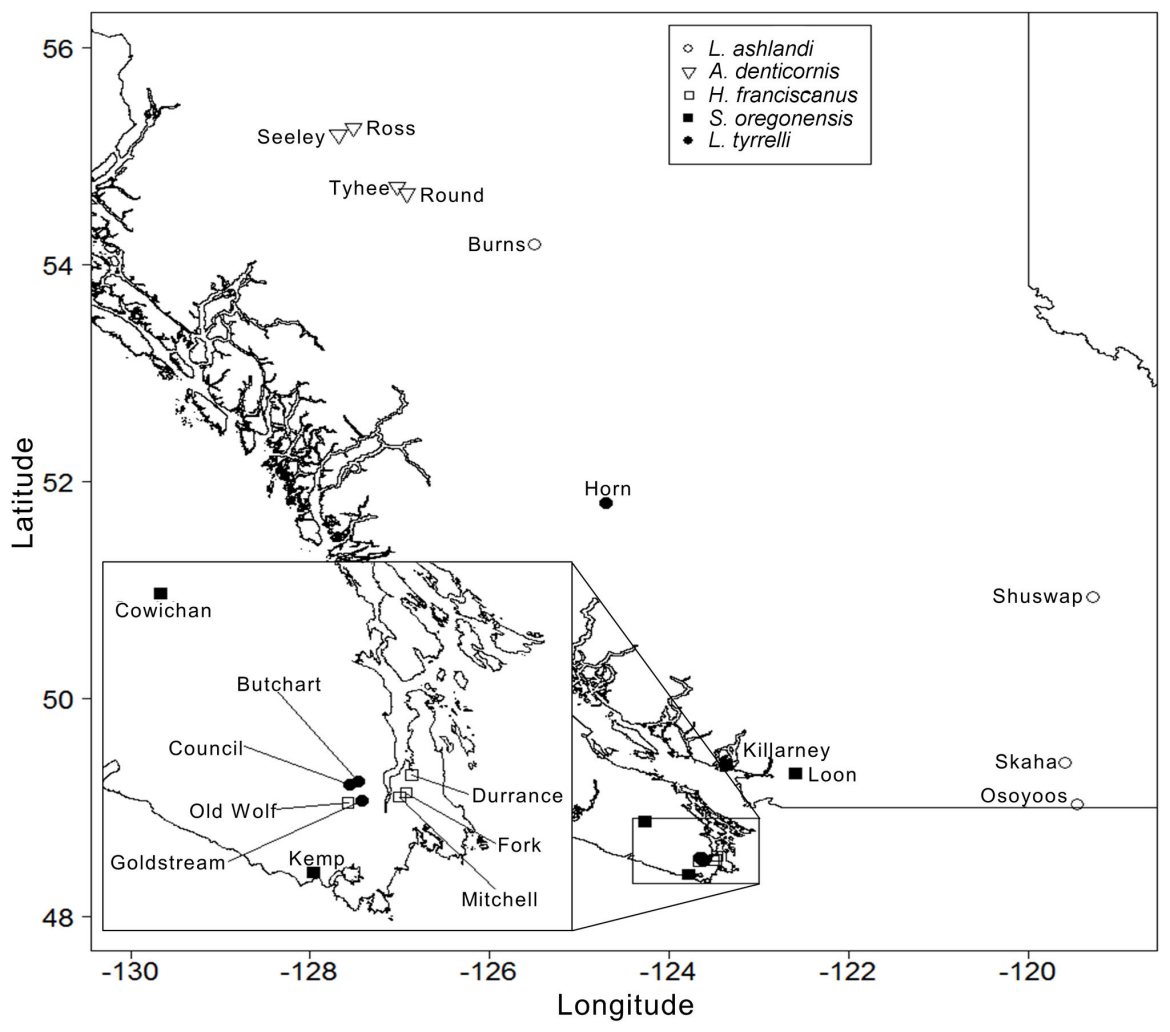
Map of sampling locations for Calanoid copepods included in the study. Base map created using the BC Watershed Atlas. Inset: Southern Vancouver Island.

 Diaptomid copepods from five species (four genera) were sampled, each from four British Columbian lakes (20 lakes total; Table 1 Figure 1). The lakes for analysis were chosen based on the general quality of sample preservation and to obtain as wide a range of lake depth and elevations within each species. Additionally, we sought to use lakes inhabited by a single Diaptomid copepod species. This was the state of the majority (but not all [27]) of lakes in this region and avoided considerations of competitive interactions among Diaptomid species or bias in species choice. To achieve this, we restricting our analysis to lakes where only a single size class of Diaptomid was observed as species pairs typically show a substantial size difference [28], putatively due to strong sexual interference among similarly sized species [29]. The geographic distributions of the species were not considered during lake selection but, except for *Leptodiaptomustyrrelli* which is also commonly found in the British Columbia Interior (near the *Leptodiaptomusashlandi* sites; Figure 1), our samples represent the central ranges of the species which tend to be limited, but overlapping, at the province scale [30]. All lakes were on public land and as they were only sampled for zooplankton (no protected species were sampled) and were largely sampled by the British Columbia Ministry of the Environment no permits were required for this work. Landmark data and associated R scripts are stored in the Dryad Digital Repository (http://dx.doi.org/10.5061/dryad.812t1).

**Table 1:** Lakes and species sampled.

**Species**	**Lake**	**Males**	**Date Sampled**	**Maximum Depth (m)**	**Area (ha)**	**Elevation (m)**
*Acanthodiaptomusdenticornis* (Wierzejski)	Seeley	Yes	Aug 19^th^ 03	3	20	306
	Ross	No	Aug 19^th^ 03	8	34	404
	Round	No	June 24^th^ 03	20	182	585
	Tyee	No	Aug 28^th^ 02	22	318	522
*Hesperodiaptomus* *franciscanus* (Lilljeborg)	Fork	Yes	June 3^rd^ 03	10	4	216
	Durrance	No	Jan 29^th^ 04	17	15	134
	Mitchell	No	April 3^rd^ 03	8	3	160
	Old Wolf	No	Mar 13^th^ 03	13	24	340
*Leptodiaptomusashlandi* (Marsh)	Shuswap	Yes	June 12^th^ 03	162	30512	347
	Burns	No	Aug 26^th^ 02	40	1180	702
	Osoyoos	No	June 25^th^ 03	63	1512	276
	Skaha	No	June 26^th^ 03	57	1959	339
*Leptodiaptomustyrrelli* (Poppe)	Goldstream	Yes	July 19^th^ 02	27	75	457
	Butchart	No	Aug 15^th^ 02	38	23	547
	Council	No	July 21^st^ 05	17	16	402
	Horn	No	June 20^th^ 03	40	171	940
*Skistodiaptomus* *oregonensis* (Lilljeborg)	Kemp	Yes	Dec 16^th^ 04	12	25	33
	Cowichan	No	July 20^th^ 03	152	6204	164
	Killarney	No	July 18^th^ 07	8	45	25
	Loon	No	Aug 8^th^ 07	58	46	344

Lake characteristics obtained from HabitatWizard [32]. See Figure 1 for lake locations. Males: whether or not males were analyzed from the lake.

We measured 5 females per lake and an additional 5 males from one lake for each species (Table 1) for a total of 125 individuals (100 females, 25 males). Males were sampled from each species, but not population, to detect general (family-level) patterns of sexual dimorphism while focusing effort on detecting intraspecific variation in a single sex. Lakes were sampled using a combination of vertical and drag tows with a fine-mesh (64 µm) zooplankton net between 2002 and 2007 and samples were frozen for analysis. Freezing and thawing was deemed to be acceptable as the copepod’s segments tended to separate rather than distort such that poorly preserved individuals could be avoided. Diaptomid copepods were identified to species based on 5^th^ leg and antennal structure using Sandercock et al. [26], and individuals were sexed using antennae and urosomes of males [31]. Mature adults were selected based on the quality of preservation and body position (lying flat with antennae projected horizontally) to facilitate shape analysis. Information on lake morphometry and elevation was obtained from HabitatWizard [32].

### Shape analysis

**Figure 2:**
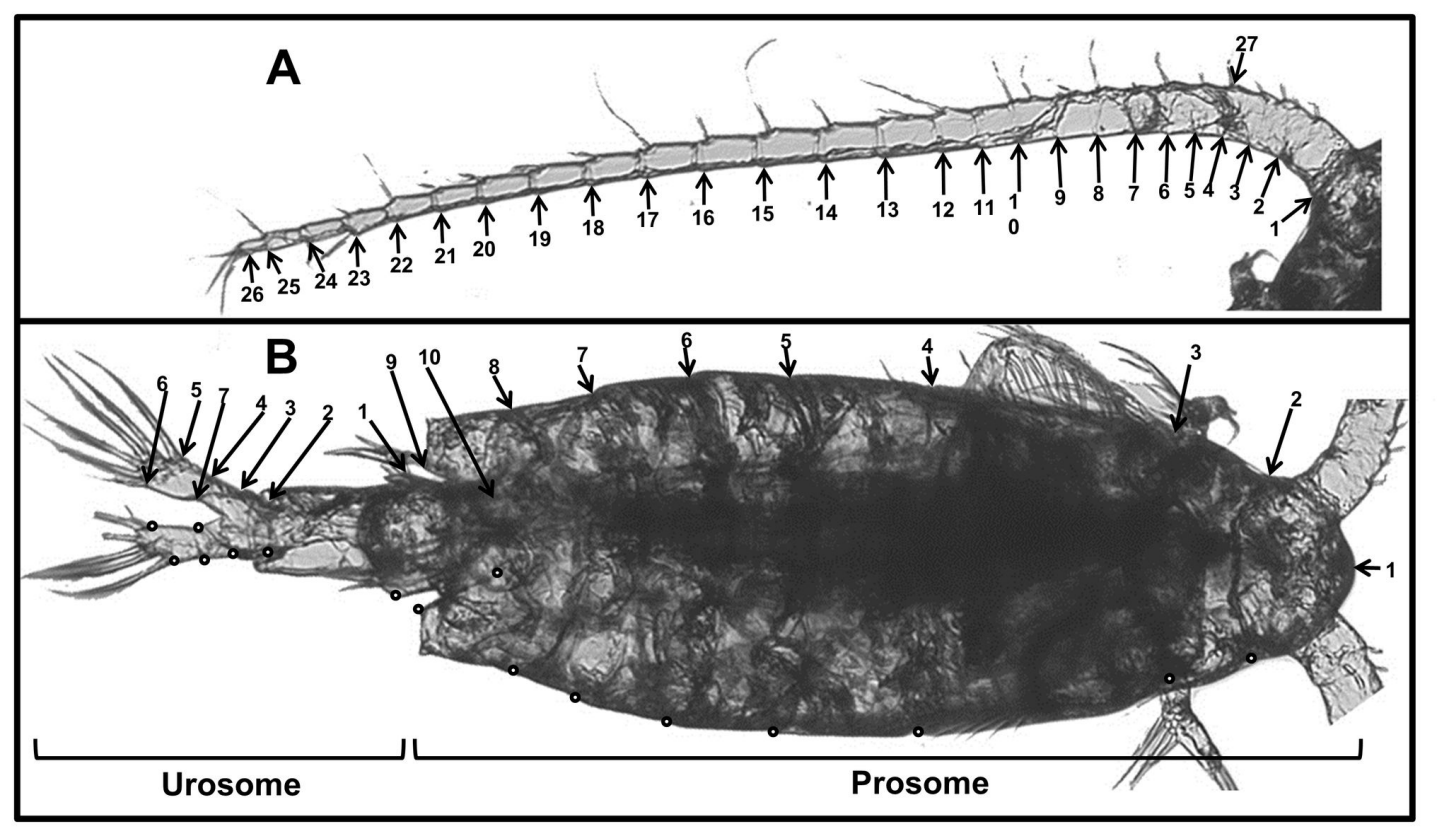
Location and designation of landmarks on the (a) antennae, (b) prosome, and urosome. Dots represent landmarks which are symmetrical to those labeled. Asymmetry was removed (see text) so only labeled landmarks were considered in the analyses. The Y-vector of prosome 9 and the X-vector of urosome 1 were not considered (see text).

Copepods were photographed dorsally using a Nikon Eclipse TE2000-S inverted microscope with attached JCV CMount Digital Camera across a range of focus heights to ensure each region of interest was in focus. Images were merged into one focused image using Auto-Montage Pro software (Version 5.03 (S), © 2005 Synoptics Inc., Frederick, Maryland). The antennae, prosome, and urosome were photographed separately to allow for increased magnification (40x for prosome and antennae, 100x for urosome) and sharper focusing. Photographs were analyzed using tpsDig software (version 2.12, © 2008 F. James Rohlf) to obtain 27, 19, and 14 landmarks for the antennae, prosome, and urosome respectively (Figure 2).

Antennae landmarks were placed at the interior base (1) and tip (26) of the antennae as well as internally at the joints between each of the 25 antennal segments on the left antennae (2 to 25) (Figure 2a). Landmark 27 was placed on the external side of the antennae at the joint between segments 3 and 4 to estimate antennal width (Figure 2a). The width was estimated at this joint as joints 1 and 2 (points 2 and 3) are less clearly defined and the width at the base is influenced by head shape and size. For consistency, joint landmarks were placed more distally when the exact joint location was ambiguous. The landmarks on the prosome were as follows (Figure 2b): (1) anterior tip of cephalosome (2,3), first and second cleavage of cephalosome (4–8), external edge of thoracic somites (9), posterior tip of body (10), internal base of fifth thoracic somite. Landmarks 2 through 10 were identified on both sides of the body. To allow prosome and urosome landmarks to be considered together, prosome landmarks 8, 9, and 10 were also located on the urosome images. In addition, the landmarks for the urosome were as follows (Figure 2b): (1) width of first abdominal somite (2–4), external edge of abdominal somites 3 through 5 (5,6), external and internal tip of furcal rami, and (7) internal base of furcal rami.

 Landmark data were processed using R statistical software [33]. Measurements were calibrated using a micrometer. To remove the influence of antennae bending, antennae landmarks were converted to cumulative segment lengths and one segment width by calculating (the cumulative sum of) the distances between adjacent landmarks. Asymmetry was removed from prosome and urosome landmarks by calculating the mean shape of the original landmarks and their mirror image for each individual using generalized Procrustes Analysis [34]. The urosome data were then rotated and transposed such that the overlapping landmarks (prosome 8-10) lined up for each individual. These points were then removed and the prosome and urosome landmarks were merged (the body). The resulting body coordinates for all individuals were superimposed using generalized Procrustes Analysis [35] with no scaling as this allowed us to keep size and allometric changes in shape together in the later principle component analysis (see below; repeating the analysis with scaling produced qualitatively similar results, identifying similar shape axes but in a different order. Notably, the smallest PC axis considered in the no-scaling analysis was the first axis in the scaled analysis). The dataset was rotated so the midline lay on the x-axis. Redundant coordinates (those not numbered in Figure 2b) were removed and the remaining coordinates were considered as variables for the analysis [34]. As the landmarks Prosome9 and Urosome1 were described only in one axis (most posterior and widest point, respectively), only the relevant vector was considered in the analysis.

Measurement error for each landmark was assessed using freshly collected samples by repositioning and re-photographing the individuals from three lakes twice. Repeatability was calculated using a nested analysis of variance as the sum of squares attributed to copepod identity divided by the total sum of squares [34], and averaged 0.94 (range: 0.72-1.00) for the body landmarks and 0.93 (range: 0.84-0.98) for antennae segment length.

### Data Analysis

Landmark coordinate data were analyzed using a Principle Component (PC) Analysis with a covariance matrix. Morphological variation across all sampled individuals was partitioned into 56 Principle Component axes, of which the first four, denoted PC_small_, PC_A:B_, PC_thin_, and PC_U:P_, were considered for analysis based on a scree test. The first four axes accounted for 93.76, 4.45, 0.56, and 0.45% of the variation in landmark position, respectively (cumulative = 99.22%). A PC analysis using only the female specimens provided nearly identical axes of interest (correlation between the loadings of corresponding axes > 0.97). A linear discriminant analysis was used to determine the degree of species differentiation described by these four axes in conjunction. As males were not sampled from all lakes, the discriminant analysis was restricted to the female samples. Predicted species identity was assigned to the species with the highest posterior probability.

Inter- and intra-specific variation for the four considered PC axes were partitioned by variance component analyses [36] using a Restricted Maximum Likelihood Estimator. We treated species and populations as random factors, and tested for each effect’s significance using likelihood ratios. As we did not perform repeated measures on the individuals used in this analysis, measurement error for each axis was estimated using the individuals from the repeatability analysis projected into the PC space from the main analysis. Within-population variation was then estimated as the residual variation minus the estimated measurement error from the repeatability analysis individuals. As we did not sample males from every lake, sex could not be included as a factor in the above analysis. Instead, paired t-tests contrasting the mean score of males and females across populations were used to identify axes showing significant sexual dimorphism.

To test for phenotype by environment correlations, we investigated the relationship between copepod shape and lake morphometry and location. Lake characteristics were summarized using the first two principle component axes of a correlation matrix of lake latitude, elevation, circularity, log maximum depth, and square root surface area. Circularity measures the complexity of the lake surface using the deviation from roundness and is calculated as the true surface area divided by the surface area of a circle with the lakes perimeter (0 = complex, 1 = round). The first two axes, size and location, explained 33 and 27% of the variation in lake characteristics, respectively. Size was positively correlated with lake area and maximum depth and negatively correlated with circularity (r = 0.57, 0.60, and -0.53, respectively). Location was positively correlated with lake latitude and elevation (r = 0.72 and 0.70 respectively). The population mean scores (females only) for each PC axis were regressed against the additive effects of lake size and location. To test if intraspecific morphological variation was shaped by the same environmental gradients as those influencing interspecific variation, the deviations of each population from its species mean for each PC axis were regressed against the deviations in lake size (Size_IS_), and location (Location_IS_). As we found similar relationships in both analyses (across all populations and across conspecifics), we further regressed the species mean PC scores against the species mean lake characteristics to confirm that intraspecific differences were not driving the “interspecific” results. Due to the very low sample size for this analysis (5 species), we restricted the regression to only the significant covariate from the all-populations analyses (size for PC_small_ and location for PC_A:B_).

## Results

### Inter and Intraspecific variation

Copepod body length varied from 0.93 to 2.06 mm, with a mean of 1.44 mm ± 0.25 and 1.34 mm ± 0.21 for males and females, respectively (mean ± SD; Table 2). On average, the urosome accounted for 28% ± 2 of the body’s length and the maximum width of the body was generally 26% ± 2 of the body length. Antennae length was approximately equal to body length (0.99% ± 0.05) for all species except the largest, *Acanthodiaptomusdenticornis* , for which it was relatively short (0.87% ± 0.04).

**Table 2:** Mean morphological trait values for males and females of each of five Diaptomid species.

Species	Sex	Body Length (μm)	Antennae:Body Length	Prosome Width:Length	Urosome:Prosome Length
*A* *. denticornis*	Female	1822 (108)	0.88 (0.01)	0.28 (0.01)	0.38 (0.01)
	Male	1656	0.82	0.27	0.40
*H* *. franciscanus*	Female	1532 (48)	0.97 (0.02)	0.26 (0.01)	0.35 (0.01)
	Male	1408	0.95	0.26	0.40
*L* *. ashlandi*	Female	1167 (157)	0.98 (0.04)	0.26 (0.02)	0.34 (0.02)
	Male	1029	1.01	0.24	0.44
*L* *. tyrrelli*	Female	1399 (75)	0.99 (0.01)	0.26 (0.00)	0.37 (0.01)
	Male	1332	0.94	0.24	0.42
*S* *. oregonensis*	Female	1269 (120)	1.05 (0.04)	0.24 (0.00)	0.41 (0.01)
	Male	1293	0.90	0.23	0.40

Means are the average of 5 individuals from a single population for males or 5 individuals from each of 4 populations for females. Values in brackets are the standard deviation of the species’ four population means showing interpopulation variation (not available for males).

**Figure 3:**
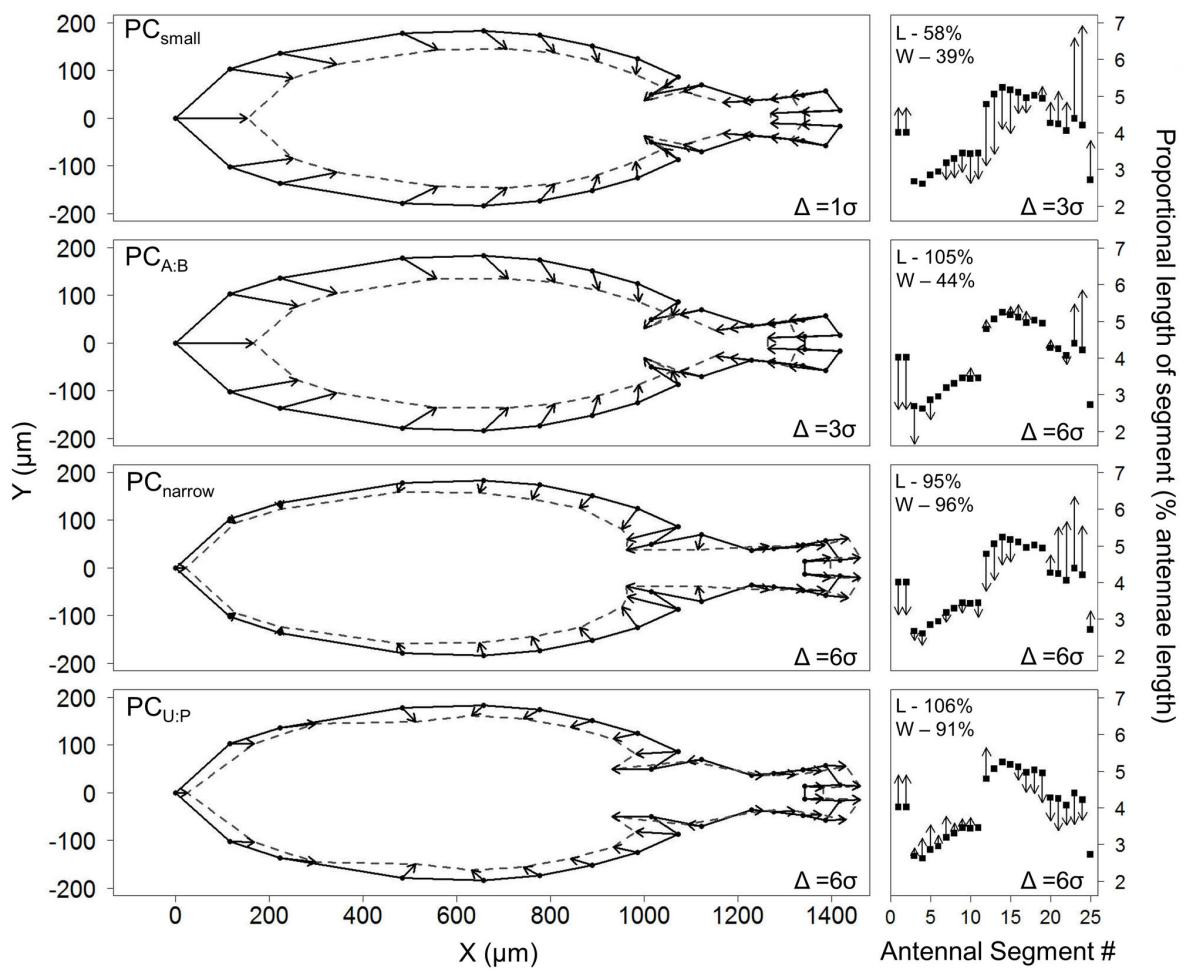
Differences in copepod shape and size as represented by the first 4 PC axes. Panels on the left and right depict changes in body shape and antennae shape respectively. Points show the mean shape across all individuals with arrows representing an increase along the PC axis (in the positive direction from the mean value) of the given number of sample standard deviations (Δ = #σ). Body: The solid line outlines the mean shape; the dashed, grey line outlines the resulting shape. Antennae: L – relative change in antennae length; W – relative change in antennae width. Arrows for changes smaller than the size of the points (0.15% antennae lengths) are not shown. The relative ratios of antennae length to body length with an increase of one sample standard deviation away from the mean for each PC axis are 103, 109, 99, and 100%, respectively.

Morphological variation associated with population identity was characterized by the first four principle component axes, PC_small_, PC_A:B_, PC_thin_, and PC_U:P_. PC_small_ characterized the size of the entire copepod (body and antennae) and allometric changes in body width (Figure 3), where larger PC_small_ scores describe relatively smaller and narrower individuals. The antennae also showed allometric changes in shape which, in conjunction with mean segment lengths, roughly defined five continuous antennal regions (Figure 3 PC_small_): Basal (segments 1-2), Proximal (3–11), Mid (12–18), Distal (19–24), and Apical (25). Smaller individuals had relatively longer distal and apical antennae segments relative to the mid and proximal segments. PC_A:B_, characterized the length of the antennae relative to the body, describing the same allometric changes in body shape as PC_small_ (the loadings for the body landmarks are highly correlated (R = 0.99) with those for PC_small_) but without a proportional change in antennae length (Figure 3).

Together, PC_small_ and PC_A:B_ describe 99.8% of body length variation (linear regression, F_2,122_ >> 100, P << 0.001). PC_narrow_ and PC_U:P_ characterized similar changes in prosome and urosome shape (Pearson’s r = 0.81). Increases in PC_narrow_ describe a narrowing and tapering of both the urosome and the prosome with a large reduction in the 5^th^ thoracic somite, a lengthening of the urosome and a correlated lengthening of the distal antennae segments relative to the mid segments (Figure 3). Increases in PC_U:P_ describe a more homogeneous decrease in the prosome and increase in the urosome with a correlated shortening of the mid and distal segments relative to the basal and proximal segments (Figure 3).

 Both species and populations accounted for a significant fraction of the variation among female Diaptomids in the first four PC axes (Table 3). Differences among species accounted for the majority of the variation in all four axes (50 to 70%; Table 3) while the variance explained by populations declined from the first to the fourth PC axis.

**Table 3:** The variance in the first four PC axes explained by differences between species and populations.

		**PC_small_**			**PC_A:B_**			**PC_narrow_**			**PC_U:P_**		
	**df**	**Var**	**%**	**P**	**Var**	**%**	**P**	**Var**	**%**	**P**	**Var**	**%**	**P**
**Species**	4	5538	65	<0.001	243	70	<0.001	27	59	<0.001	14	50	<0.001
**Population**	15	1986	23	<0.001	57	16	<0.001	6	13	<0.001	3	9	0.038
**Individual**	80	929	11		26	8		5	12		7	24	
**Measurement**		54	1		22	6		7	16		5	17	

Species and Population were treated as random factors. Variance components calculated using a Restricted Maximum Likelihood Estimator. Measurement error variance was calculated as the variation attributed to picture and measurement using the individuals from the repeatability analysis (see text). Individual variance is estimated as the residual variance in the species-population analysis minus the measurement error variance. Var: variance attributed to the factor; %: percent of variance explained; P: likelihood ratio test significance estimate.

**Figure 4:**
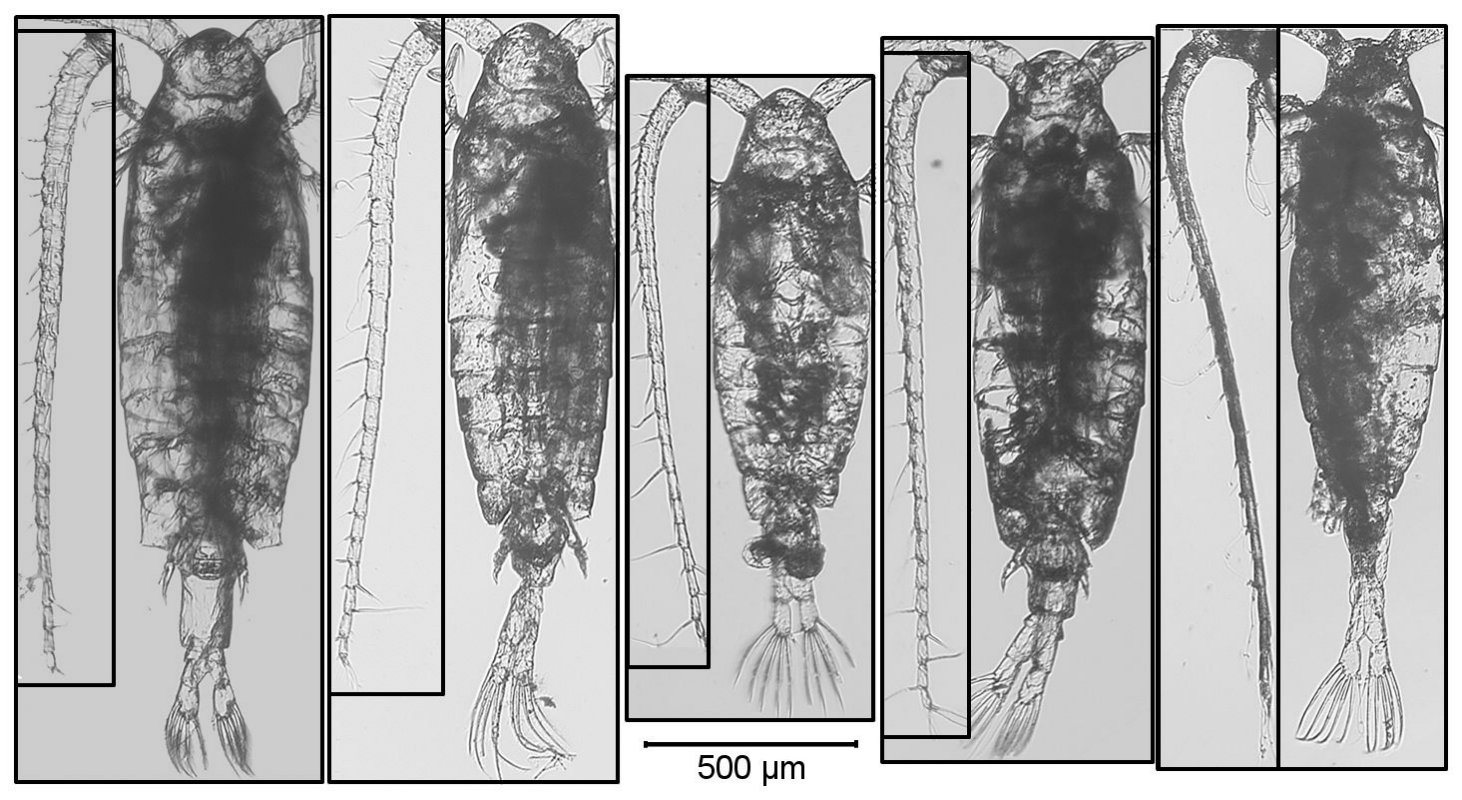
Representative females (Body and left antennae) of each of the 5 copepod species considered. Left to right: *A* *. denticornis* (Tyee Lake), *H* *. franciscanus* (Durrance Lake), *L* *. ashlandi* (Osoyoos Lake), *L* *. tyrrelli* (Council Lake), *S.* oregonensis (Kemp Lake).

No single PC axis was sufficient to differentiate among all of the species (Figure S1). Based on the linear discriminant analysis, the four axes clearly differentiated between the five species (correct assignment based on posterior probability > 0.95) except for *L* *. tyrrelli* and *Hesperodiaptomus* *franciscanus* (correct assignment of 0.75 and 0.80, respectively) which were morphologically more similar. Briefly, *L* *. ashlandi* is small with relatively small urosomes, *A* *. denticornis* is large with relatively short antennae, *H* *. franciscanus* and *L* *. tyrrelli* are narrow-bodied, while *Skistodiaptomus* *oregonensis* is wide-bodied with relatively long antennae (Figure 4).

### Sexual dimorphism

 Male copepods were consistently smaller than females (Pairwise t-test, PC_small_, T_4_ = -4.3, P = 0.012) and had shorter antennae relative to their body size (PC_A:B_, T_4_ = -3.0, P = 0.042). On average, males were 92% as long as females and had an antennae length of approximately 92% of their body length (versus 97% for females). The sexes did not show consistent differences in PC_narrow_ (T_4_ = -1.9, P = 0.13) or PC_U:P_ (T_4_ = -2.0, P = 0.012) across species.

### Lake size and location

 The size and geographic location (latitude and elevation) of lakes explained 59% of the between-population variation in female copepod’s PC_small_ and PC_A:B_ scores (Table 4). Mean body size decreased with increasing lake depth and, across all species except *A* *. denticornis* (the largest and most northerly species), mean body size also decreased with increasing latitude and elevation (F_2,13_ = 16, R^2^ = 0.72, P < 0.001, P[Location] = 0.001). The decline in body size with lake size and location was also observed among populations of the same species. Body size similarly decreased relative to each species’ mean with increasing lake size and location, and these two gradients accounted for 53% of the intraspecific variation in body size (Table 4). Similarly, relative antennae length (PC_A:B_) showed a strong location effect across populations and species with comparable results observed among conspecific populations. Across all populations, relative antennae length significantly decreased with increasing latitude and elevation (Table 4). Across conspecific populations, relative antennae length decreased with latitude and elevation and increased with lake size, though only with marginal statistical significance (P = 0.052 and 0.044, respectively; Table 4). Species mean body size and relative antennae length similarly decreased with species mean lake size and location, respectively (PC_small_: F_1,3_ = 25, R = 0.89, P = 0.015; PC_A:B_: F_1,3_ = 12, R = 0.80, P = 0.041), confirming that the similarities between the inter- and intraspecific trends for PC_small_ and PC_A:B_ are not statistical artifacts.

**Table 4:** Correlations between lake morphology and elevation and mean population scores for body and antennae PC axes.

**Response**	**Model**	**F-statistic**	**P-value**	**R^2^**
**PC_small_**	**+ Size** – Location	F_2,17_ = 12.3	**<0.001**	0.59
**PC_A:B_**	- Size – **Location**	F_2,17_ = 12.0	**<0.001**	0.59
**PC_narrow_**	+ Size – Location	F_2,17_ = 0.1	0.934	0.01
**PC_U:P_**	- Size – Location	F_2,17_ = 0.9	0.407	0.10
**PC_IS_small_**	**+ Size_IS_ + Location_IS_**	F_2,17_ = 9.5	**0.002**	0.53
**PC_IS_A:B_**	- **Size_IS_** – *Location*_*IS*_	F_2,17_ = 5.2	**0.017**	0.38
**PC_IS_narrow_**	- Size_IS_ + Location_IS_	F_2,17_ = 0.2	0.980	0.00
**PC _IS_U:P_**	**+ Size_IS_ – Location_IS_**	F_2,17_ = 5.8	**0.012**	0.40

The covariates Size and Location are the first two principle components of a matrix of lake latitude, elevation, circularity, log maximum depth, and square root surface area. Size is positively correlated with depth and area and negatively correlated with circularity (r 0.60, 0.57, and - 0.53, respectively). Location is positively correlated with latitude and elevation (r 0.72 and 0.70 respectively). IS – difference from the variable’s intraspecific mean value. +/- represents direction of effect; Italics: P_effect_ < 0.1, Bold: P_effect_<0.05.

Lake size and location did not account for a significant amount of the variation in PC_narrow_ or PC_U:P_ across all populations (Table 4). However, at the intraspecific level, we found that variation in PC_U:P_, but not PC_narrow_, could be explained by both abiotic gradients considered (Table 4). Populations in larger lakes at lower elevations and latitudes had relatively longer urosomes for their body size as compared to other populations of the same species (Table 4).

## Discussion

 Previous studies have characterized Diaptomid copepods as relatively undifferentiated morphologically, despite deep genetic divergence times (10–20 Mya) [22], implying ecological neutrality of the different species [21] and a lack of functional variation with respect to potential impacts on ecosystem processes. However, our morphometric analysis revealed measurable difference in shape and size among species and populations and confirmed well-known patterns of size dimorphism between sexes (Gilbert and Williamson [37] found that Diaptomid females are 1.14 times longer than males on average (83 species, sd = 0.093, range = 0.77-1.39; our ratio = 1.08)). Population of origin explained between 18 and 35% as much morphological variation as species identity, and was always a significant predictor of morphological variation (Table 3), indicating that intra-specific variation is substantial compared to that between species. For some traits, morphological variation was correlated with environmental characteristics, including latitude and lake depth, suggesting that there potentially has been local adaptation to contrasting environments. Our results suggest that these species and populations are morphologically differentiated in traits that might underlie previously observed variation in their contrasting ecosystem effects [25], but the mechanistic links between trait variation and ecosystem effects remain unknown.

### Functional significance of morphological variation

 The multivariate analysis revealed composite morphological traits related to size (PC _small_), antennae/body length (PC_A:B_), body width (PC_narrow_) and urosome/prosome length (PC_U:P_) that showed substantial variation among species and/or populations. These traits illustrate some of the important components of body size and shape variation, and reveal some of the allometric interactions between different body components. For instance, the urosome and prosome both decrease with increasing PC_small_ and PC_narrow_, but the urosome increases relative to the prosome with increasing PC_U:P_ across species and populations (Figure 3). Such covariation helps identify functional traits suggesting, for instance, that the size of the urosome has a function beyond that of overall body size.

#### *Antennae*

A novel finding of this work is that allometric changes in antennae segment lengths suggest that the 25 segments can be grouped into 5 contiguous units: Basal (1,2), Proximal (3–11), Mid (12–18), Distal (19–24), Apical (25; Figure 3 PC_small_). These groupings appear to have an anatomical and functional basis: the basal segments are specialized to direct the antennae laterally while the mid-distal joint and the distal-apical joint coincide with the attachment points of the antennal musculature [38]. While a functional difference between the proximal and the mid units is not apparent, this suggests a possible biomechanical connection. The antennae of copepods have been strongly implicated in the mechanoreception of prey items [39,40] but little work has considered the influence of antennae shape on prey detection. The proximal region of the antennae is thought to be associated with the detection of motile prey due to the higher density of large seta [41]. As larger copepods are more likely to consume larger and more motile prey, this is consistent with our observations that the antennae length of larger bodied copepods were composed of relatively larger proximal segments (PC_small_). Landry and Fagerness [40] studied the correlation between morphological variation and prey size variation in species of marine copepods. They found that only body size and not antennae size was correlated with prey size, and only across five copepod species (2 of the initial 7 were excluded from their analysis). A multiple regression of antennae and body length against prey length identified significantly positive and negative effects of body and antennae length, respectively, on prey length across all seven species (F_2,4_ = 5.7, P = 0.068). These findings indirectly suggest that *A* *. denticornis* , which has a large body size but relatively short antennae, may be specialized to detect and target the hydrodynamic disturbances created by larger, more motile prey. This is consistent with previous gut analyses of natural *A* *. denticornis* populations that have found a large proportion of rotifers, a larger and motile prey than the phytoplankton consumed by Diaptomids in this size class [42]. While speculative, these multiple lines of evidence are in agreement and, given the paucity of research relating functional morphology with diet in copepods, we urge further studies to test for relationships between morphology and diet variation at the individual level.

#### *Body shape*

Our results suggest that morphological differences characterized by PC_small_ and PC_A:B_ varied similarly, but independently, across species, populations, and sexes. PC_small_ and PC_A:B_, together characterized body size in addition to allometric shape changes and relative antennae length, respectively. Both of these axes showed substantial variation across both species and populations relative to that within populations (65 vs. 23% for PC_small_ and 70 vs. 16% for PC_A:B_; Table 3). PC_small_ and PC_A:B_ also exhibited sexual dimorphism across all species. As with most copepods, males were consistently smaller than females, an adaptation generally attributed to a decreased relationship between body size and fecundity relative to females [37,43]. Males also had relatively short (left) antennae, a trait which may be associated with the geniculate right antennae’s secondary sexual role of grasping the female during copulation [38].

Body width varied among species with relative little variation among conspecific populations but it did not correlate with any of the measured environmental gradients. This relationship, high interspecific to intraspecific variation and no apparent abiotic associations, may be indicative of a role in reproductive isolation. This hypothesis is supported by the morphology characterized by PC_narrow_. A key characteristic of PC_narrow_ is a change in the size and shape of the 5^th^ thoracic segment and the trunk of the urosome, going from highly squared and projected in *A* *. denticornis* to reduced and tapered in *S* *. oregonensis* (Figure 3). Due the importance of this region in copulation (the male’s 5th swimming leg is heavily specialized for grasping the female’s urosome) [44] proper matching between males and females is likely important for fertilization success and variation across species may act as a prezygotic barrier to reproduction [29]. The hypothesis of a role for PC_narrow_ in reproductive isolation, though, is opposed by the similarity between *H* *. franciscanus* and *L* *. tyrrelli* in PC_narrow_ as they are the two species with the most strongly overlapping ranges in our sample (Figure 1). PC_U:P_ may have a similar sexual function, characterizing a unique sexual differentiation of *L* *. ashlandi* and, in general, our morphological analysis could be useful for identifying traits that underlie reproductive isolation between species.

#### *Phenotype by environment correlation*

Diaptomid copepods were smallest in larger lakes and there was a latitudinal and altitudinal decrease in body size across all species except *A* *. denticornis* . Diaptomid copepods at higher elevations and latitudes also had shorter antennae for their body size (Table 4). Remarkably, populations within species also followed this same pattern: copepods in deeper lakes and at higher latitudes and elevations were small and had proportionally short antennae relative to their species mean (Table 4). Such correlations between PC_small_ and PC_A:B_ with environmental conditions and geographic locations suggest either that divergent selection pressures have led to parallel patterns of adaptive differentiation across species (i.e. parallel patterns of local adaptation), or that the plastic responses are conserved across species and lead to a parallel pattern of morphological differentiation across broad environmental gradients. These alternative hypotheses can only be distinguished by common garden experiments to separate the genetic from the plastic components of morphological variation.

The relationships we observed between copepod body size and lake size and location are likely influenced by a wide variety of environmental gradients, including nutrient levels, temperature conditions, and predation pressure. From studies of copepod body size along altitudinal and latitudinal gradient it is apparent that copepods are generally smaller at higher temperatures [45,46]. Zooplankton body size also reflects an important balance between resource competition and predator avoidance [45]. Smaller individuals more adept at avoiding larger, visual predators [47] while larger individuals are better at avoiding tactile and hydrodynamic sensing predators [45]. In addition, larger individuals may be more efficient resource competitors [48], such that varying levels of food quantity and/or quality may influence body size distributions [45]. It is not clear how these multiple effects interact across depth or latitudinal/elevation gradients and unfortunately we have no information about the variation in predation pressure from fish and invertebrates among our study lakes. Interestingly, our observation that Diaptomid body size generally decreases with lake latitude, elevation, and depth is contrary to the simple models of temperature and predation which predict that zooplankton body size decreases with temperature, increasing with latitude, elevation, and lake depth [45,49], and decreases with visual predation threat, which is also expected to decrease with lake depth due to the presence of piscivores and a larger deep water refuge. As such, the multivariate phenotypic diversification we have observed will hopefully inspire future studies to quantify the multidimensional aspects of niche variation for copepods in the pelagic habitat of lakes.

### Consequences of population-level morphological variation

Little effort has been made, for copepods or any other taxonomic groups, to quantify morphological variation among populations relative to that among species. This is surprising given that both interspecific and intraspecific diversity has important effects on species coexistence and ecosystem dynamics [2,25]. While intraspecific diversity is maintained both within and between populations, comparisons of inter to intraspecific diversity appear to have focused exclusively on comparing diversity between species to that within local populations [50]. We found that, across the four composite traits that we identified, the majority of variation in population means was due to species differences, but there were significant differences between conspecific populations. Such differences accounted for an average of 20% of the population-level variation across the four morphological traits (or, interpopulation diversity was one quarder interspecific diversity) ranging from 26% for the first axis, PC_Small_, to 16% for the fourth axis, PC_U:P_. This interpopulation diversity was just over twice as large as the intrapopulation diversity for the two body size axes (PC_small_ and PC_A:B_) but was equal to and smaller than intrapopulation diversity for the shape axes PC_narrow_ and PC_U:P_, respectively. Large inter-population diversity in morphology is one possible explanation for why different populations can have contrasting ecosystem effects [25]. However, this assumes a positive relationship between phenotypic disparity and the sizes of contrasting ecosystem-effects among organisms, and this idea has never been tested directly.

It is unclear how general this relative partitioning of intra and interspecific morphological variation is across taxa. Recent work suggests that interpopulation morphological variation may be relatively high in Diaptomid copepods due to their limited dispersal ability. Leibold et al. [51] found that calanoid copepod species, in contrast to the more vagile daphniids, were sorted primarily by historical biogeography rather than local conditions. As such, habitat filtering and local adaptation is expected to have occurred more at the population level in calanoids, relative to daphniids and other more mobile species.

Apart from ours, we know of no other study that has partitioned morphological variation among species and populations, though one previous study has collected the relevant data. Chambel et al. [52] presented growth data for populations and species of Mediterranean pine grown under experimental conditions, and were interested in differences between species in both their means and their degree of intraspecific variation rather than the overall level of variation among species and populations. Applying our variance partitioning analysis to their population-level dataset we found that the diversity among conspecific populations accounted for 35% of the variation among all populations (Range: 9–79%). This result was strongly influenced by an outlier population (PR-LE); without this population, conspecific population differences accounted for 20% of the variation (Range: 5–36%). In our study, we too found that conspecific population differences accounted for 20% of the variation among populations (Range: 16–26%) suggesting, though very tentatively, that Diaptomid copepods have a relatively normal partitioning of phenotypic variation between species and conspecific populations. Further studies comparing variation among species and populations will be useful for revealing the functional consequences of the partitioning of morphological variation within and among species.

## Conclusions

We found that while the morphological differences between Diaptomid species are subtle to the observer, variation among species and populations is detectable using morphometrics and is correlated with environmental and geographic gradients. These findings suggest that while the Diaptomid copepod radiation has shown relative morphological stasis in broad characteristics of the body plan, there is significant variation present between both populations and species that might be relevant for understanding the causes and consequences of phenotypic evolution in this group of species.

## Supporting Information

Figure S1 Clustering of female Diaptomids along the four principle component axes of body shape by species and populations. Species are identified by color and bounded by a convex hull: Red - *A* *. denticornis* , Green - *H* *. franciscanus* , Black - *L* *. ashlandi* ; Purple - *L* *. tyrrelli* ; Blue - *S* *. oregonensis* . Populations within each species are denoted by different symbols. The same symbol across heterospecific populations does not imply association. See text and Figure 3 for a description of the four axes, PC_small_, PC_A:B_, PC_narrow_, and PC_U:P_.
(TIF)
