# Supplementary figures and images for: Variation in Body Shape across Species and Populations in a Radiation of Diaptomid Copepods
Source: PLoS One. 2013 Jun 27;8(6):e68272. doi: 10.1371/journal.pone.0068272 (PMC3694880; doi:10.1371/journal.pone.0068272)

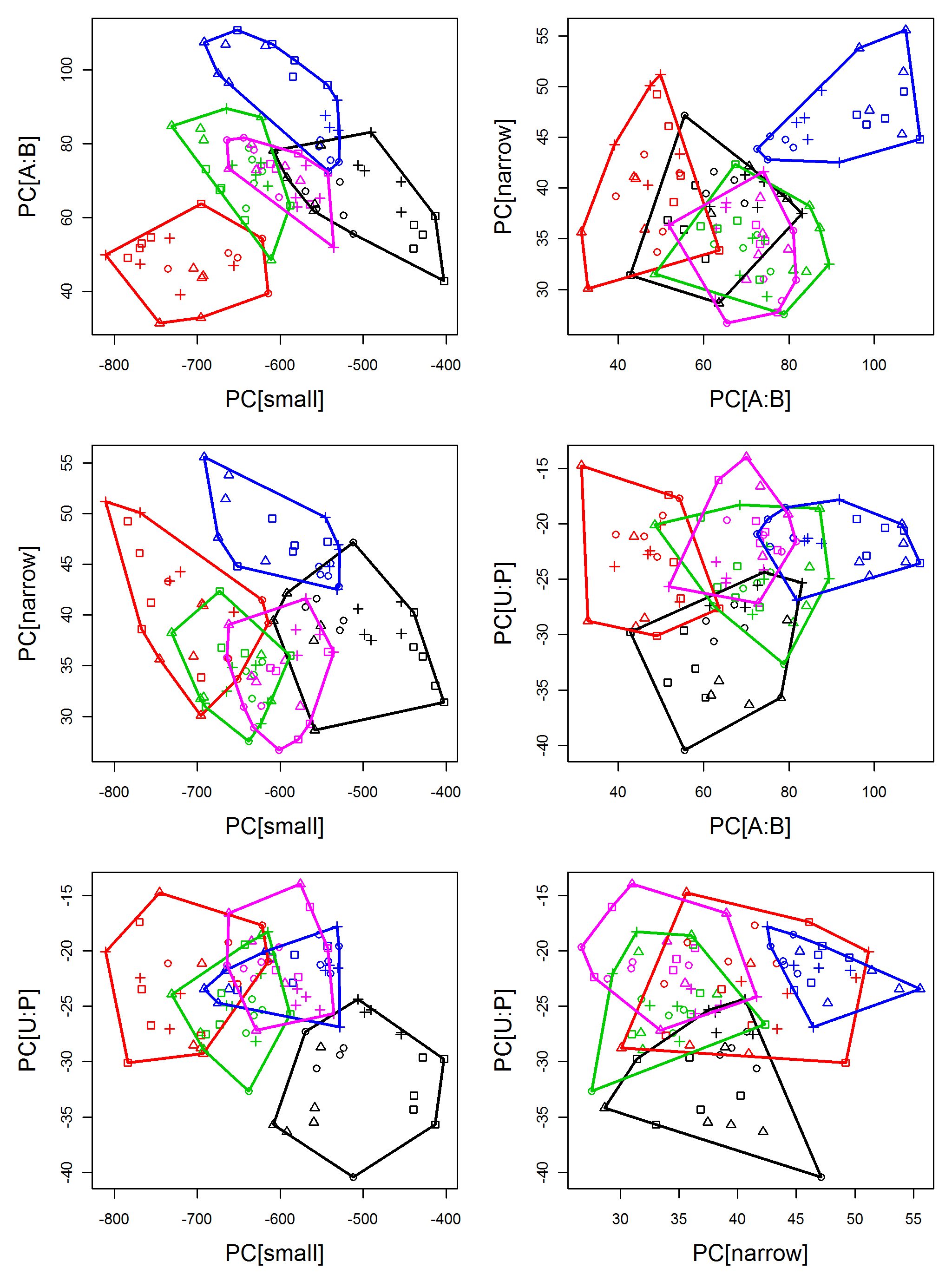

Supplement: Figure S1 — Species are identified by color and bounded by a convex hull: Red - A . denticornis , Green - H . franciscanus , Black - L . ashlandi ; Purple - L . tyrrelli ; Blue - S . oregonensis . Populations within each species are denoted by different symbols. The same symbol across heterospecific populations does not imply association. See text and Figure 3 for a description of the four axes, PCsmall, PCA:B, PCnarrow, and PCU:P. (TIF) [file pone.0068272.s001.tif]
